# Audio-motor and related audio-spatial interventions for spatial function in people with visual impairment: a systematic review

**DOI:** 10.3389/fpsyg.2026.1927485

**Published:** 2026-10-26

**Authors:** Jianda Hu, Qinran Yu, Mengting Zou, Shuo Ma, Danyang Zhu

**Affiliations:** 1 School of Mathematical Sciences, Zhejiang Normal University, Jinhua, China; 2 School of Education and Psychology, Tianjin University of Sport, Tianjin, China; 3 Key Laboratory of Psychological and Physiological Regulation of Competitive Sports, General Administration of Sport, Tianjin, China; 4 College of Physical Education and Health Sciences, Zhejiang Normal University, Jinhua, China; 5 School of Exercise and Nutrition Sciences, Queensland University of Technology, Brisbane, QLD, Australia; 6 College of Psychology, Zhejiang Normal University, Jinhua, China

**Keywords:** audio-motor training, audio-spatial intervention, auditory localization, auditory sensory substitution, navigation, spatial cognition, systematic review, visual impairment

## Abstract

**Background:**

Auditory feedback linked to movement or active spatial exploration may support non-visual spatial learning, but the evidence spans conceptually distinct training, assistive-technology, and sensory-substitution approaches.

**Objective:**

To systematically review audio-motor and related audio-spatial interventions for auditory localization, proprioception, spatial perception, spatial cognition, mobility, and navigation in people with visual impairment.

**Methods:**

Web of Science, Scopus, and EBSCOhost were searched from database inception to December 2024. A targeted supplementary search through 15 July 2026 was used to identify recent contextual studies and emerging technologies, but was not treated as a complete *de novo* update of the principal systematic search. Peer-reviewed intervention studies involving participants with visual impairment were eligible; sighted participants were permitted only as comparator groups. Risk of bias was assessed with RoB 2 for studies reported as randomized and ROBINS-I for non-randomized studies. Because populations, intervention mechanisms, study designs, outcomes, measurement instruments, and reporting formats were heterogeneous, findings were synthesized narratively following the Synthesis Without Meta-analysis (SWiM) framework.

**Results:**

Ten studies involving 151 participants were included in the principal synthesis. Interventions comprised movement-contingent auditory devices, spatial-audio or game-based navigation training, audiovisual motor training, and camera-based auditory feedback. Findings suggested possible improvements in selected task-specific outcomes, particularly auditory localization, reaching or proprioceptive matching, and virtual or real-world navigation. Standardized effect sizes were presented when reported, but effects were inconsistent and several studies relied on small samples, within-group comparisons, or non-randomized designs.

**Conclusions:**

The certainty of evidence was low or very low across outcome domains. Preliminary evidence suggests that selected audio-motor and related audio-spatial interventions may improve specific spatial tasks in some visually impaired populations, but heterogeneity, imprecision, and risk of bias preclude firm conclusions regarding clinical effectiveness, optimal dose, long-term benefit, or prevention of developmental delay.

## 1 Introduction

Vision impairment includes a wide spectrum of reduced near or distance vision and should not be treated as synonymous with blindness. The World Health Organization estimated that at least 2.2 billion people worldwide have near or distance vision impairment, while the severity, cause, age at onset, and functional consequences vary substantially across individuals (WHO, 2019a,b). Vision contributes to postural control and the rapid updating of body position relative to environmental features; when visual information is unavailable or unreliable, people may depend more strongly on auditory, tactile, vestibular, and proprioceptive information (Russo et al., 2017; Shams and Seitz, 2008; Aggius-Vella et al., 2019).

Spatial function is a multidimensional construct. In this review, auditory localization refers to identifying the direction or distance of a sound source; proprioception refers to information about body or limb position and movement; spatial perception refers to the online estimation of spatial relations; spatial representation refers to an internal representation of locations or layouts (Chen et al., 2016); spatial cognition includes higher-order learning, memory, and manipulation of spatial information; and navigation refers to route planning and movement through virtual or real environments. These domains are related but are not interchangeable (Linn and Petersen, 1985; Vasilyeva and Lourenco, 2012).

For the purposes of this review, audio-motor training was operationally defined as training in which auditory feedback is systematically and temporally linked to a participant's own movement or goal-directed motor action. Audio-spatial training was defined more broadly as active training using spatialized auditory information to support localization, spatial learning, or navigation, without necessarily mapping every sound to movement. Audiovisual motor training combines visual and auditory feedback linked to action, while sensory-substitution systems transform non-auditory environmental information, such as camera input, into auditory cues. Audio games and virtual navigation systems were treated as distinct intervention categories when their primary mechanism was active spatial learning rather than movement-contingent feedback (Taiyu, 2019). In the title and review question, “related audio-spatial interventions” is therefore used as an umbrella term, while these four intervention categories remain conceptually and analytically distinct.

Experimental work indicates that movement and body representation can influence auditory spatial perception, supporting a plausible link between sensorimotor experience and auditory spatial coding (Paromov et al., 2024). In visually impaired populations, devices such as the Audio Bracelet for Blind Interaction (ABBI), the Audio-based Environment Simulator (AbES), audiovisual motor-training systems, and camera-based spatial-audio systems have been evaluated for outcomes including localization, proprioception, mobility, and navigation (Merabet et al., 2012; Connors et al., 2014; Cappagli et al., 2017b, 2019; Cuppone et al., 2019; Ahmad et al., 2021; Hu et al., 2022). Recent peer-reviewed work has also expanded toward multimodal wearable navigation systems combining auditory cues with computer vision or haptic feedback in blind and visually impaired users (Wang and Kuchenbecker, 2012; Tang et al., 2025; Skulimowski et al., 2025; Han et al., 2026; Lucio-Naranjo et al., 2026). These developments reinforce the need to distinguish interventions intended to produce rehabilitation or retained spatial learning from assistive systems that provide moment-to-moment guidance, even when both use spatial auditory cues. The diversity of mechanisms and outcome domains makes broad claims about “spatial improvement” difficult to interpret.

Previous studies have often been discussed together despite important differences in visual condition, age, intervention type, comparator, and outcome measure. Congenital or early-onset blindness may differ from acquired blindness or low vision in developmental experience and adaptive plasticity (Pasqualotto and Proulx, 2012; Röder et al., 2004; Senna et al., 2024), and evidence from sighted participants under simulated visual deprivation cannot be assumed to generalize directly to clinical populations. A systematic synthesis therefore requires an explicit taxonomy and outcome-specific interpretation.

This systematic review aimed to characterize audio-motor and related audio-spatial interventions for people with visual impairment and to examine their reported effects across six prespecified domains: auditory localization, proprioception, spatial perception, spatial representation, spatial cognition, and navigation or mobility. Secondary objectives were to compare findings by population, age group, study design, and intervention category; evaluate risk of bias separately for randomized and non-randomized studies; and identify priorities for future research. The review was designed as a systematic review with structured narrative synthesis rather than a meta-analysis.

## 2 Methods

This review was reported in accordance with PRISMA 2020 and the PRISMA-S extension for literature searches (Page et al., 2021; Rethlefsen et al., 2021). Because quantitative pooling was not appropriate, the synthesis was additionally structured according to the SWiM reporting guideline and Cochrane guidance for reviews without meta-analysis (Campbell et al., 2020; McKenzie and Brennan, 2024). The review was not prospectively registered, and no publicly accessible protocol was prepared.

### 2.1 Review question and operational definitions

The review question was: In people with visual impairment, what effects have audio-motor or related active audio-spatial interventions shown on auditory localization, proprioception, spatial perception, spatial representation, spatial cognition, mobility, or navigation? Studies were assigned to one of four intervention categories before synthesis: (1) movement-contingent audio-motor training; (2) active audio-spatial or game-based navigation training; (3) audiovisual motor training; and (4) camera-based or sensory-substitution feedback. Outcomes were grouped according to the functional definitions provided in the Introduction.

### 2.2 Eligibility criteria

Eligible studies were peer-reviewed intervention studies published in English that: (1) included participants with diagnosed blindness, low vision, retinal disease, or visual-field loss; (2) evaluated an intervention in one of the four categories defined above; (3) reported at least one prespecified spatial outcome; and (4) used a randomized, non-randomized controlled, quasi-experimental, single-group pre-post, pilot, or case-study design. Sighted participants were eligible only as comparator groups and were not treated as equivalent to visually impaired participants. Studies involving additional neurological or motor conditions were retained only when visual impairment was present and were interpreted separately. Reviews, comments, dissertations, abstracts without a full report, and studies without extractable intervention outcomes were excluded. Conference proceedings were excluded because many reports did not provide sufficient methodological or outcome detail for risk-of-bias assessment; this decision may have introduced selection bias and is acknowledged as a limitation.

### 2.3 Information sources and search strategy

Web of Science Core Collection, Scopus, and EBSCOhost were searched from database inception to December 2024. The search used three concept blocks: (1) visual impairment, visual disability, blindness, or low vision; (2) audio, auditory, music, sound, or sound sources; and (3) spatial cognition or space cognition. These concepts were expanded during revision to include audio-motor, audio-spatial, auditory localization, proprioception, mobility, navigation, sonification, and sensory substitution. A representative master string was: (blind^*^ OR “visual impair^*^” OR “visual disabilit^*^” OR “low vision” OR scotoma^*^) AND (audio OR auditory OR music OR sound OR “sound source^*^” OR “audio-motor” OR “auditory-motor” OR “spatial audio” OR “audio-spatial” OR “audio game^*^” OR sonification OR “sensory substitution”) AND (“spatial perception” OR “auditory localization” OR propriocept^*^ OR “spatial cognition” OR “spatial representation” OR orientation OR mobility OR navigation). Database-specific search strings are provided in Supplementary Table S1. Reference lists of included studies were screened, and no automation tools were used. A targeted supplementary search through 15 July 2026 applied the expanded concept blocks across PubMed/MEDLINE, targeted IEEE- and ACM-indexed records, publisher and index pages, and preprint servers to identify recent contextual literature and emerging technologies. Because this supplementary search was not a complete *de novo* update with full deduplication and duplicate independent screening across all sources, it was not used to alter the principal included-study set; this limitation is stated explicitly below.

### 2.4 Selection process

After deduplication in EndNote, two reviewers independently screened titles and abstracts and then independently assessed potentially eligible full texts. Disagreements were resolved by discussion and, when necessary, consultation with a third reviewer. Inter-rater agreement was not calculated. Full-text reports were excluded when they did not provide analyzable intervention data, did not provide sufficient methodological information to establish eligibility, or were published outside the prespecified English-language restriction. Risk of bias was assessed separately after study inclusion.

### 2.5 Data extraction and data items

One reviewer extracted data using a standardized form and a second reviewer checked all entries against the source report. Extracted variables included country, study design, sample size, age, visual condition and age at onset, comparator, intervention category and components, dose and duration, follow-up, outcome domain, measurement instrument, numerical result where reported, and information required for risk-of-bias assessment. Disagreements were resolved by consensus. Study authors were not contacted for missing data; unavailable data were recorded as not reported.

### 2.6 Risk-of-bias assessment

Two reviewers assessed risk of bias independently, with disagreements resolved by discussion or consultation with a third reviewer. Study-design classifications and methodological information were checked against the full-text reports. Only Ahmad et al. (2021) and Merabet et al. (2012) explicitly reported randomized allocation and were assessed with RoB 2 (Sterne et al., 2019). The remaining eight studies were assessed with ROBINS-I (Sterne et al., 2016) because they used non-randomized controlled, single-group pre-post, comparative usability, pilot, or single-case designs. RoB 2 and ROBINS-I judgments are presented separately and were not combined numerically. For RoB 2, an overall judgment of “some concerns” was assigned when at least one domain raised some concerns and no domain was judged high risk. For ROBINS-I, the overall judgment reflected the most severe domain: serious overall risk was assigned when at least one domain was serious and none was critical, while any critical domain produced a critical overall judgment. Brief study-specific rationales for the overall judgments are provided in Tables 1, 2A, 2B.

**Table 1:** ROBINS-I judgments for non-randomized studies (panel A).

ROBINS-I domain	Connors et al. (2014)	Cappagli et al. (2017b)	Bǎlan et al. (2017)	Cappagli et al. (2019)
Bias due to confounding	Serious	Serious	Serious	Serious
Bias in selection of participants	Moderate	Moderate	Moderate	Moderate
Bias in classification of interventions	Low	Low	Low	Low
Bias due to deviations from intended interventions	Moderate	Moderate	Moderate	Moderate
Bias due to missing data	Low	Low	Low	Moderate
Bias in measurement of outcomes	Moderate	Moderate	Moderate	Moderate
Bias in selection of reported result	Moderate	Moderate	Serious	Moderate
Overall judgment	Serious	Serious	Serious	Serious
Primary rationale for overall judgment	No untreated comparator and a very small single-group sample; findings were vulnerable to confounding, practice effects, and selective reporting.	Non-randomized pre-post design with very small visual-impairment subgroups and sighted baseline comparators rather than an equivalent untreated group.	Single-group pre-post design without a parallel comparator, with serious concerns about confounding and selective reporting.	Center-linked non-random allocation, possible baseline confounding, incomplete follow-up, and outcome-measurement concerns.

**Table 2A:** ROBINS-I judgments for non-randomized studies (panel B).

ROBINS-I domain	Cuppone et al. (2019)	Martolini et al. (2022)	Tinelli et al. (2022)	Hu et al. (2022)
Bias due to confounding	Serious	Serious	Critical	Serious
Bias in selection of participants	Moderate	Serious	Critical	Serious
Bias in classification of interventions	Low	Low	Low	Low
Bias due to deviations from intended interventions	Moderate	Moderate	Moderate	Moderate
Bias due to missing data	Low	Low	Low	Low
Bias in measurement of outcomes	Moderate	Moderate	Serious	Moderate
Bias in selection of reported result	Moderate	Moderate	Serious	Moderate
Overall judgment	Serious	Serious	Critical	Serious
Primary rationale for overall judgment	No untreated control; within-group pre-post changes were vulnerable to time, practice, and regression-to-the-mean effects.	Only three participants per group, non-random allocation, and baseline imbalance created serious confounding and selection concerns.	Single-case design with major confounding and selection limitations and no concurrent comparator, producing a critical overall judgment.	Small non-randomized usability study with blindfolded sighted comparators; outcomes mainly reflected immediate device-task performance.

**Table 2B:** RoB 2 judgments for randomized studies.

ROBINS-I domain	Ahmad et al. (2021)	Merabet et al. (2012)
Bias arising from the randomization process	Some concerns	Some concerns
Bias due to deviations from intended interventions	Some concerns	Some concerns
Bias due to missing outcome data	Low	Some concerns
Bias in measurement of the outcome	Some concerns	Some concerns
Bias in selection of the reported result	Some concerns	Some concerns
Overall judgment	Some concerns	Some concerns
Primary rationale for overall judgment	Randomization and allocation procedures, assessor blinding, and prospective analysis plans were incompletely described, but no domain was judged high risk.	Allocation concealment, missing-data handling, assessor blinding, and prospective analysis plans were incompletely reported, but no domain was judged high risk.

### 2.7 Synthesis methods and certainty of evidence

A meta-analysis was not undertaken because the studies differed in visual conditions, age groups, intervention mechanisms, comparators, study designs, outcome definitions, measurement instruments, follow-up periods, and the availability of numerical data. No clinically coherent outcome subgroup contained enough comparable effect estimates and variance information for reliable pooling. The previously described “effectiveness rate” based on the number of statistically significant trials was removed because vote counting by statistical significance can be misleading. Following SWiM, each study was first assigned to one of the four prespecified intervention categories and then to one or more functional outcome domains. Within each domain, randomized or controlled between-group evidence was presented before uncontrolled pre-post change and task-performance evidence. Findings were compared according to study design, comparator, direction and magnitude of effect, precision, and risk of bias. Standardized effect sizes were extracted and reported when the source study provided them; they were not calculated when the report lacked sufficient variance or group-level information. Immediate device-assisted performance was distinguished from retained learning or transfer beyond the trained task. Certainty was judged qualitatively for each outcome domain using risk of bias, inconsistency, indirectness, imprecision, and potential publication bias.

## 3 Results

### 3.1 Study selection

The original database searches identified 1,829 records, and three additional records were identified through reference-list searching. After removal of 625 duplicates, 1,207 records remained. Title and abstract screening excluded 1,181 records, leaving 26 reports for full-text assessment. Sixteen full-text reports were excluded: nine did not report data suitable for analysis, four did not provide sufficient methodological information to establish eligibility, and three were non-English reports. Ten studies were included in the principal narrative synthesis (Figure 1). The targeted supplementary search through 15 July 2026 identified recent peer-reviewed assistive-device evaluations and emerging preprints that were used to update the background and discussion. Because this was not a complete *de novo* systematic update, these later reports were not integrated into the principal study-selection counts or risk-of-bias synthesis.

**Figure 1:**
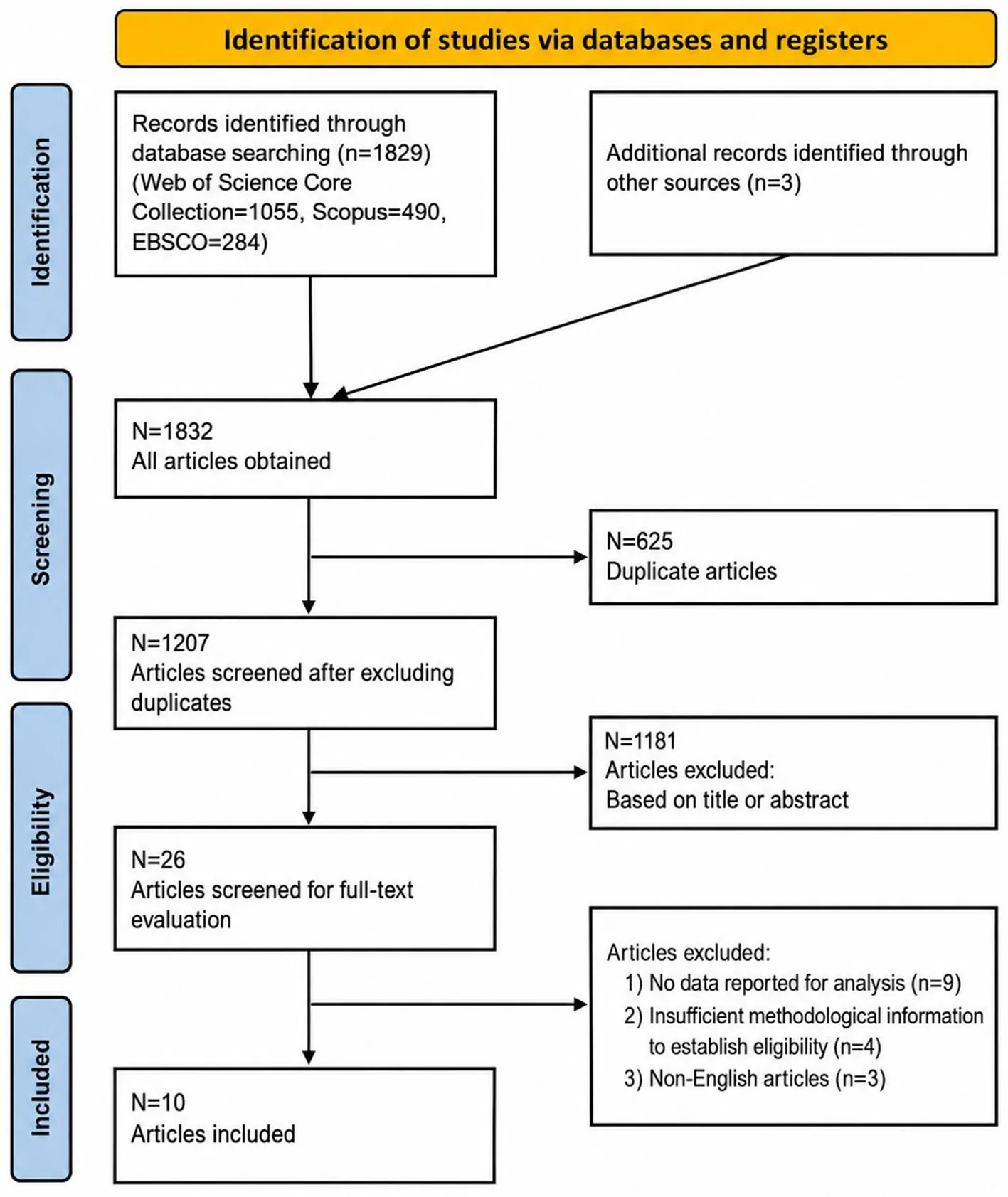
Flow chart for literature retrieval.

### 3.2 Characteristics of included studies

The 10 included studies were published between 2012 and 2022 and involved 151 participants. Across the reports, 126 participants had visual impairment, including one child with hemianopia and an additional motor disorder, and 25 were sighted comparison participants. Studies were conducted in Italy (*n* = 6), the United States (*n* = 2), China (*n* = 1), and Romania (*n* = 1). Two studies explicitly reported randomized allocation (Ahmad et al., 2021; Merabet et al., 2012). The remaining studies included non-randomized controlled comparisons, single-group pre-post studies, a pilot transfer study, a within-subject comparative usability study, and a single-case feasibility study.

### 3.3 Population and intervention taxonomy

Participants ranged from approximately 3 to 85 years of age and included young children, adolescents, adults, and older adults. Visual conditions included congenital or early-onset blindness, acquired or long-term late blindness, low vision, retinal degenerative disease with scotoma, residual vision, and hemianopia. One single-case study involved hemianopia with hemiplegia and was interpreted separately. Sighted participants served as baseline or modality-comparison groups and were not treated as clinically equivalent to visually impaired participants. Interventions were classified as movement-contingent audio-motor training (principally ABBI), active audio-spatial or game-based navigation training (principally AbES or 3D audio games), audiovisual motor training, or camera-based spatial-audio feedback.

### 3.4 Risk of bias

Risk-of-bias findings are presented separately for the two randomized studies and eight non-randomized studies (Tables 1, 2A, 2B). Both randomized studies were judged to raise some concerns, primarily because allocation concealment, assessor blinding, missing-data handling, and prospective analysis plans were incompletely described. The non-randomized studies were judged to have serious risk of bias overall, except the single-case study, which was judged critical, chiefly because of confounding, participant selection, absent or non-equivalent comparators, small samples, and incomplete prespecification. Study-specific rationales are shown directly beneath the domain judgments in Tables 1, 2A, 2B. These judgments reduce confidence in causal interpretation.

### 3.5 Intervention dose and outcome measures

Intervention dose varied from a single session of approximately 10 min to programs lasting 12 weeks. Session frequency, duration, supervised practice, and home practice were inconsistently reported. Outcomes included auditory localization and distance judgments, auditory or proprioceptive reaching, spatial representation, mobility, virtual or real-world navigation, game performance, and task-specific interaction measures. Follow-up beyond the immediate post-intervention assessment was uncommon, limiting conclusions about retention and transfer.

### 3.6 Narrative synthesis by outcome domain

The findings below distinguish randomized or controlled between-group evidence from uncontrolled within-group change and task performance. Studies were grouped first by intervention mechanism and then by functional outcome domain, with stronger comparative designs discussed before uncontrolled evidence within each domain. Owing to heterogeneous measures and incomplete numerical reporting, effect estimates could not be pooled. Selected numerical findings and standardized effect sizes available in the included full reports are reported in Table 3. Where a source report did not provide an estimate, standardized effect size, or measure of precision, the table states NR rather than inferring a value.

**Table 3:** Characteristics of included studies.

Study (year, country)	Population and sample	Study design/comparator	Intervention category and dose	Outcome instruments	Outcome domain	Selected numerical finding and interpretation
Connors et al. (2014), United States	*n* = 7 early-blind adolescents (3 male); mean age 16.5 years; blindness documented before age 3	Single-group pilot/transfer study; all participants received AbES; assessment order, not intervention, was randomized	15-min familiarization followed by 60-min AbES game play	Ten direct-route trials and ten exit-route trials in the real building	Navigation and spatial learning	Direct-route success was 70.00% ± 12.91%; exit-route success was 97.14% ± 4.88%; shortest-path selection was 71.43% ± 38.48%. Game score correlated with direct-route performance (*r* = 0.845, *p* = 0.017) and exit-route performance (*r* = 0.787, *p* = 0.036). No untreated comparator was included.
Cappagli et al. (2017b), Italy	Seven visually impaired children (2 blind, mean age 3.5 years; 5 low vision, mean age 4.4 years) plus 14 sighted baseline comparators (mean age 3.6 years)	Non-randomized pre-post intervention in visually impaired children; sighted participants served as a baseline comparator	ABBI, 1 h/week for 3 months	Static and dynamic auditory localization error	Spatial perception/auditory localization	Blind children showed reduced localization error in static (*Z* = −2.8, *p* < 0.01) and dynamic tasks (*Z* = −2.4, *p* < 0.01). Low-vision children showed no clear improvement (dynamic *p* = 0.60; static *p* = 0.05). Findings are subgroup-specific and based on very small samples.
Ahmad et al. (2021), Italy	*n* = 22 with retinal degenerative disease and central scotoma; training *n* = 11 (mean age 67.27 ± 17.16), control *n* = 11 (68.00 ± 19.93)	Randomized matched parallel-group study; brief audiovisual-motor training vs. rest/repeated testing	Single brief AVT/IVATT session between pre- and post-tests; total experimental visit approximately 2 h	Auditory and visual localization across central and peripheral regions	Auditory localization and spatial representation	For auditory localization, the group × phase interaction was significant (*F*_(1, 20)_ = 6.68, *p* = 0.01, generalized η^2^ = 0.01). Effects were modest and varied by region and outcome.
Merabet et al. (2012), United States	*n* = 17 early-blind adults; randomized to gamer or directed-navigation learning	Randomized parallel comparison of two active AbES learning strategies	Three 90-min training sessions	Room-to-room route finding and shortest-exit “drop-off” task	Navigation and spatial cognition	Direct-route success was similar (87.5% ± 10.4 vs. 88.57% ± 18.6; *p* = 0.89). Gamers selected shorter exits more often (2.50 ± 0.65 vs. 1.25 ± 0.00 points; *t* = 5.03, *p* = 0.0002), while navigation time did not differ (*p* = 0.59).
Cappagli et al. (2019), Italy	*n* = 44 visually impaired children aged 6–17; ABBI *n* = 22 and classical rehabilitation *n* = 22; main pre-post analyses included 18 and 20, respectively	Non-randomized controlled study; allocation was linked to rehabilitation center/program rather than reported randomization	12 weeks: 45 min/week supervised plus ~5 h/week home practice (69 h total); 1-year follow-up in 10 ABBI participants	Auditory localization, bisection, distance, auditory/proprioceptive reaching, and mobility	Spatial cognition, proprioception, and mobility	Between-group ANCOVA favored ABBI for auditory localization (*F*_(1, 32)_ = 38.29, *p* = 0.0001, *d* = 1.45), bisection (*p* = 0.0005), auditory reaching (*p* = 0.0005), proprioceptive reaching (*p* = 0.01), and mobility (*p* = 0.04), but not auditory distance (*p* = 0.62). Follow-up was limited to 10 trained children.
Cuppone et al. (2019), Italy	*n* = 16: 9 early-blind adults (41 ± 15 years) and 7 sighted adults (32 ± 4 years); both groups received training	Non-randomized pre-post comparison by visual status; no untreated control group	10-min audio-motor training in four 2.5-min blocks	Auditory reaching and ipsilateral joint-position matching	Auditory localization and proprioception	Auditory matching error decreased after training (time effect *F* = 14.27, *p* = 0.0004; blind subgroup *t*(8) = 5.9, *p* = 0.0003). Proprioceptive error also decreased (time effect *F* = 17.16, *p* = 0.0001; blind subgroup *t*(8) = 4.9, *p* < 0.01). Group × time interactions were not significant.
Martolini et al. (2022), Italy	*n* = 6 adults with long-term late blindness, aged 47–62; training *n* = 3, no-training control *n* = 3	Small non-randomized controlled longitudinal study; random allocation was not reported	4 weeks: 1 h/week supervised plus 1–3 h/day home activities (mean approximately 42 h)	Horizontal/vertical auditory localization, proprioceptive matching and midline, spatial bisection, EEG/ERP	Spatial representation and proprioception	Horizontal localization did not improve (group × session *p* = 0.90). Vertical localization showed group × session effects for relative and absolute error (χ^2^ = 22.2 and 13.4, both *p* < 0.001). Proprioceptive matching improved (χ^2^ = 5.3, *p* = 0.02); the sample was only three per group and baseline imbalance was noted.
Tinelli et al. (2022), Italy	Single child with hemianopia, severe nystagmus, and hemiplegia	Single-case feasibility study with pre-post and 1-year observations	ABBI for 2 weeks: 1 h/day therapist-led plus 1 h/day home training	Teddy bear cancellation test, motor visual search, assisting hand assessment, actigraphy	Visual-spatial attention and upper-limb function	Teddy Bear omissions decreased from 4 to 1 and were 0 at 1 year. AHA increased from 7 to 22 units and was 16 units at 1 year. These data demonstrate feasibility in one child and are not generalizable.
Hu et al. (2022), China	*n* = 7: 3 participants with congenital blindness (22.6 ± 1.5 years) and 4 blindfolded sighted participants (25.5 ± 4.2 years)	Non-randomized within-subject comparative usability study of four feedback strategies	Single-session Fitts-law and desktop-manipulation experiments using StereoPilot	Information-transfer rate, positioning error, completion time, success rate, and desktop object localization	Camera-based spatial-audio feedback/target localization	For visually impaired participants, spatial-audio rendering produced an information-transfer rate nearly three times that of voice and vibrotactile feedback; positioning error was 28% lower than voice, 39% lower than vibration, and 46% lower than non-speech audio. Evidence concerns task/device performance, not rehabilitation training.
Bǎlan et al. (2017), Romania	*n* = 10 visually impaired adults (5 women, 5 men), aged 27–63; residual vision 0%−15%	Single-group pre-post multimodal audio-game study	Two-day protocol with auditory-haptic localization training and 3D audio-game testing	Navigation deviation, correct travel decisions, completion time, and obstacle hits	Audio-game navigation and sound localization	Correct travel decisions increased from 74.3% to 82.1% (*t* = 3.4, *p* ≤ 0.05). Completion time decreased from 32.5 to 25.9 s, but this change was not statistically significant. No parallel control group was included.

ABBI, audio bracelet for blind interaction; AbES, audio-based environment simulator; AVT, audiovisual motor training; NR, not reported. Study-design labels and selected numerical findings were checked against the full-text reports. Values are reported as published. Standardized effect sizes (including *r*, Cohen's *d*, and generalized η^2^) are shown when the source report provided them; NR is used when an estimate or measure of precision was unavailable, and no effect size was inferred without sufficient group-level or variance data.

#### 3.6.1 Navigation and mobility

AbES and audio-game studies reported that blind participants could learn virtual layouts and transfer some route knowledge to real-world or simulated navigation tasks (Merabet et al., 2012; Connors et al., 2014; Bǎlan et al., 2017). Controlled comparisons were limited, and some findings were based on success rates or post-training task performance rather than a direct between-group effect. These studies therefore provide preliminary evidence of task-specific navigation learning, but not firm evidence of generalized clinical mobility benefit.

#### 3.6.2 Auditory localization and spatial representation

Audiovisual motor training and movement-contingent auditory training were associated with improved localization or reaching accuracy in some studies (Ahmad et al., 2021; Martolini et al., 2022). However, findings varied by spatial dimension and task. In the late-blind sample studied by Martolini et al. (2022), improvement was reported for vertical localization, whereas horizontal localization did not show a comparable group-level change. The evidence therefore suggests possible domain-specific recalibration rather than a uniform improvement in auditory spatial representation.

#### 3.6.3 Proprioception and spatial perception

ABBI-based studies reported reduced auditory or proprioceptive matching error and improvements on selected static or dynamic spatial tasks in visually impaired children and adults (Cappagli et al., 2017a,b; Cuppone et al., 2019). The interpretation depends on whether the reported analysis compared intervention and control groups or only pre-post performance within the trained group. Because samples were small and measures differed across studies, the magnitude and generalizability of benefit remain uncertain.

#### 3.6.4 Broader spatial cognition and technology-mediated feedback

Broader spatial-cognition outcomes were reported in studies using ABBI, camera-based spatial-audio feedback, and multisensory devices (Cappagli et al., 2019; Hu et al., 2022; Tinelli et al., 2022). These studies suggest that active auditory feedback may support specific spatial tasks or device-mediated interaction. Nevertheless, the evidence includes heterogeneous designs and one single-case feasibility study, so it cannot establish effects on global spatial cognition, independence, quality of life, or social participation.

### 3.7 Qualitative certainty of evidence

Certainty was judged as low or very low across outcome domains. Risk of bias was a major concern, results were inconsistent across tasks, populations and interventions were indirect for any single clinical recommendation, sample sizes were imprecise, and publication bias could not be formally assessed because of the small number and heterogeneity of studies. The evidence supports cautious hypotheses about task-specific benefit but not definitive estimates of clinical effectiveness.

## 4 Discussion

This review identified a small and heterogeneous evidence base evaluating movement-contingent auditory feedback, audio-spatial games, audiovisual motor training, and camera-based auditory systems. Across these categories, several studies reported improvements in selected localization, reaching, or navigation tasks. The pattern is compatible with the idea that active sensorimotor experience can recalibrate non-visual spatial representations, but the evidence does not show that all auditory interventions act through the same mechanism or improve all aspects of spatial function.

Movement-contingent systems such as ABBI provide a particularly direct test of audio-motor coupling because sounds are linked to the user's action. Audio-game and virtual-navigation systems instead emphasize active exploration and spatial learning. Audiovisual motor systems couple action with more than one sensory channel, whereas camera-based systems constitute sensory substitution because environmental information is transformed into sound. These categories should remain conceptually distinct even though they are grouped under the broader term “related audio-spatial interventions” in the title. Indirect experimental evidence that body representation influences auditory spatial perception supports a mechanistic link between action and auditory space, but it does not by itself demonstrate rehabilitation effectiveness in visual impairment (Paromov et al., 2024).

The targeted supplementary search illustrates how quickly the field is expanding beyond the studies in the principal synthesis. Recent peer-reviewed systems combine spatial audio with wearable computer vision, haptic feedback, or multimodal training and have been evaluated with blind or visually impaired users (Tang et al., 2025; Skulimowski et al., 2025; Han et al., 2026; Lucio-Naranjo et al., 2026). Emerging preprints have additionally described mixed-reality navigation, geometry-aware generative spatial audio, and standardized sound-first training scenarios (Pfitzer et al., 2025; Muñoz, 2026; Luo et al., 2026). These reports are relevant to the direction of the field but differ in intervention mechanism, evaluation purpose, publication status, or timing relative to the principal search. They were therefore used as contextual evidence rather than incorporated into the principal synthesis. A future fully updated review should screen these newer records systematically and determine whether they meet a prospectively specified distinction between rehabilitation, retained learning, and moment-to-moment assistive performance.

The findings were more persuasive when they involved a controlled between-group comparison than when they reflected only pre-post improvement within a trained group. A statistically significant change in an intervention group, combined with a non-significant change in a control group, is not equivalent to a significant between-group difference. Accordingly, the revised synthesis avoids causal language where an interaction or direct group contrast was not reported.

Age and age at onset may plausibly influence spatial learning and adaptive plasticity, but the included evidence did not directly compare early with later intervention or establish that early audio-motor training prevents developmental delay. Early intervention should therefore be considered a theoretically motivated research priority rather than a confirmed clinical recommendation. Future studies should prespecify developmental subgroups and report age at onset, residual vision, baseline mobility, and prior rehabilitation experience.

Current evidence is insufficient to determine an optimal intervention intensity, session duration, or total training dose. Claims regarding quality of life, emotional regulation, social integration, independence, or long-term functional benefit were not directly tested by most included studies and have therefore been removed. Future trials should include patient-relevant mobility and participation outcomes, longer follow-up, adverse-event reporting, and measures of transfer beyond the trained task.

For practice, the findings justify continued development and carefully monitored evaluation of accessible auditory technologies, but not routine recommendation of a single device or protocol. Occupational therapists and orientation-and-mobility professionals may consider these technologies as adjuncts for clearly defined, task-specific goals such as reaching, sound localization, route learning, or safe environmental exploration. Selection should follow individualized assessment of residual vision, hearing, motor function, cognitive load, device tolerance, and the person's daily environments. Training should begin in graded and controlled settings, document baseline and post-training performance, monitor fatigue or adverse events, and verify transfer to meaningful daily activities. Auditory technologies should complement rather than replace established mobility aids and individualized rehabilitation. For research, adequately powered trials should compare clearly defined intervention mechanisms, use active comparators, report complete numerical results, standardized effect sizes and confidence intervals, and distinguish auditory localization, proprioception, spatial perception, spatial cognition, and navigation as separate outcomes.

The principal contribution of this review is therefore conceptual and methodological: it organizes a diverse literature into intervention and outcome categories and identifies where preliminary findings exist. The review does not establish a pooled treatment effect. The targeted supplementary search through 15 July 2026 strengthened the contextual discussion but was not a complete *de novo* update and therefore did not alter the principal included-study set. Future updates should maintain broad coverage of psychology, rehabilitation, engineering, and human-computer-interaction sources, apply prospectively specified eligibility criteria to recent multimodal systems, and reassess whether sufficiently comparable outcome subsets support quantitative synthesis.

The overall interpretation must remain cautious because the evidence base comprises only 10 small studies with heterogeneous methods and incomplete reporting. The absence of a meta-analysis is not itself a limitation; the key limitations are the lack of comparable effect estimates, limited controlled evidence, and uncertainty about bias and generalizability.

### 4.1 Limitations

This review has several limitations. The primary review-level limitation is that the review was not prospectively registered and no publicly accessible protocol was prepared. This reduces transparency about prespecified eligibility, outcome grouping, and synthesis decisions and increases the possibility of *post hoc* methodological changes. Second, the original systematic search was limited to Web of Science, Scopus, and EBSCOhost through December 2024. The targeted supplementary search through 15 July 2026 covered PubMed/MEDLINE, selected engineering and human-computer-interaction records, publisher and index pages, and preprint servers, but it was not a complete *de novo* update with full duplicate screening and risk-of-bias assessment. Accordingly, recent contextual reports were not incorporated into the principal synthesis, and missed studies, publication bias, and selection bias remain possible. Third, Embase, CINAHL, CENTRAL, PsycINFO as a standalone database, non-English reports, and the full conference literature were not comprehensively searched. Fourth, interventions, populations, study designs, outcomes, and follow-up periods were heterogeneous, while numerical effect estimates, standardized effect sizes, and measures of precision were incompletely reported. Fifth, several studies had small samples, non-randomized or uncontrolled designs, or serious risk-of-bias concerns. Finally, no formal review-level assessment of reporting bias was feasible, certainty was assessed qualitatively rather than with a full GRADE procedure, and the sighted comparator groups and one participant with an additional motor disorder were not directly representative of the broader visually impaired population.

## 5 Conclusions

Ten small and methodologically heterogeneous studies evaluated movement-contingent audio-motor devices, audio-spatial or game-based navigation, audiovisual motor training, and camera-based auditory feedback in visually impaired populations. Preliminary findings indicate possible improvements in selected task-specific outcomes, including auditory localization, proprioceptive or auditory reaching, and virtual or real-world navigation. The certainty of evidence was low or very low, and confidence was limited by risk of bias, imprecision, heterogeneous interventions and measures, and limited controlled evidence. Consequently, no firm recommendation can be made regarding clinical effectiveness, optimal dose, long-term benefit, or prevention of developmental delay. Future research should prioritize adequately powered randomized controlled trials using prospectively registered protocols, standardized and patient-relevant outcome measures, complete reporting of effect sizes and precision, active comparators, and longer follow-up periods to assess retention, transfer, and real-world functional benefit.
