# Supplementary material for: Audio-motor and related audio-spatial interventions for spatial function in people with visual impairment: a systematic review
Source: Front Psychol. 2026 Oct 26;17:1927485. doi: 10.3389/fpsyg.2026.1927485 (PMC13745482; doi:10.3389/fpsyg.2026.1927485)
Supplement: Supplementary file 1 [file Table_1.docx]

**Supplementary Material**

*Audio-motor and related audio-spatial interventions for spatial function in people with visual impairment: a systematic review*

**Supplementary Table S1. Database-specific search strategies**

| **Source** | **Search string / approach** | **Limits and date range** |
| --- | --- | --- |
| Web of Science Core Collection | TS=(("visual impairment*" OR "visual disabilit*" OR blind* OR "low vision" OR scotoma*) AND (audio OR auditory OR music OR sound OR "sound source*" OR "audio-motor" OR "auditory-motor" OR "spatial audio" OR "audio-spatial" OR "audio game*" OR sonification OR "sensory substitution") AND ("spatial perception" OR "auditory localization" OR propriocept* OR "spatial cognition" OR "spatial representation" OR orientation OR mobility OR navigation)) | English-language peer-reviewed articles; inception to December 2024. |
| Scopus | TITLE-ABS-KEY(("visual impairment*" OR "visual disabilit*" OR blind* OR "low vision" OR scotoma*) AND (audio OR auditory OR music OR sound OR "sound source*" OR "audio-motor" OR "auditory-motor" OR "spatial audio" OR "audio-spatial" OR "audio game*" OR sonification OR "sensory substitution") AND ("spatial perception" OR "auditory localization" OR propriocept* OR "spatial cognition" OR "spatial representation" OR orientation OR mobility OR navigation)) | English-language articles; inception to December 2024. |
| EBSCOhost | TX (("visual impairment*" OR "visual disabilit*" OR blind* OR "low vision" OR scotoma*) AND (audio OR auditory OR music OR sound OR "sound source*" OR "audio-motor" OR "auditory-motor" OR "spatial audio" OR "audio-spatial" OR "audio game*" OR sonification OR "sensory substitution") AND ("spatial perception" OR "auditory localization" OR propriocept* OR "spatial cognition" OR "spatial representation" OR orientation OR mobility OR navigation)) | English and peer-reviewed limits; inception to December 2024. |
| Targeted supplementary update through 15 July 2026 | Expanded concept blocks applied to PubMed/MEDLINE, targeted IEEE- and ACM-indexed records, publisher/index pages, and preprint servers to identify recent contextual literature and emerging technologies. | Targeted search after December 2024 through 15 July 2026; not a complete de novo update and not incorporated into the principal study-selection counts. |
